# Posterior pedicle screw fixation to treat lower cervical fractures associated with ankylosing spondylitis: a retrospective study of 35 cases

**DOI:** 10.1186/s12891-017-1396-5

**Published:** 2017-02-14

**Authors:** Liang Yan, Zhenguo Luo, Baorong He, Jijun Liu, Dingjun Hao

**Affiliations:** 1 Department of Spinal Surgery, Hong Hui Hospital, Xi’an Jiaotong University College of Medicine, 555 Friendship East Road, Xi’an, 710054 Shaanxi China; 2 Department of Anesthesiology, Hong Hui Hospital, Xi’an Jiaotong University College of Medicine, 555 Friendship East Road, Xi’an, 710054 Shaanxi China

**Keywords:** Ankylosing spondylitis, Cervical, Fracture, Pedicle screw, Posterior

## Abstract

**Background:**

The most common site of fractures in patients with ankylosing spondylitis (AS) is the cervical spine, especially the lower cervical spine and cervicothoracic junction. The optimal treatment for cervical spine fractures secondary to AS is controversial. This study aimed to explore the effects of posterior pedicle screw fixation alone on fractures of the lower cervical spine in patients with AS.

**Methods:**

From January 2006 to January 2013, a total of 35 patients with AS and a lower cervical spine fracture were treated using only posterior cervical/thoracic pedicle screw fixation. In this retrospective study, we reviewed the patients’ charts to assess their case histories, operations, neurological outcomes, and complications. We also evaluated their postoperative radiographs to determine the time of bone fusion.

**Results:**

Altogether, 32 (91.4%) of the 35 fractures resulted from an acute injury and 3 (8.6%) from a chronic injury. In 25 cases, the fracture resulted from a low-energy spinal injury and in 8 cases from a high-energy injury. Posterior pedicle screw fixation was successful in all patients, with radiographic fusion confirmed by computed tomography. The average time of bone fusion was 3.6 months (range 3 − 6 months). The surgery improved the American Spinal Injury Association grade in 15 (42.9%) patients. No intraoperative complications occurred. None of the corrections resulted in neurological decompensation. The average postoperative correction was 18°.

**Conclusions:**

Pedicle screw fixation and autologous bone grafting through a single posterior approach to lower cervical spine fractures in AS patients could stabilize the spine, correct kyphosis, and relieve pressure. It is thus reasonable to recommend this surgical strategy for AS-associated fractures of the lower cervical spine.

**Trial registration:**

Not applicable.

## Background

Ankylosing spondylitis (AS) is a seronegative, progressive, systemic, inflammatory, rheumatic spondyloarthropathy that mainly affects the spine and sacroiliac joints [1]. The condition is associated with progressive ossification of the spinal ligaments and ankylosis of the facet joints, eventually leading to a completely stiff spine. Calcification of the longitudinal ligaments eventually results in the classic radiographic “bamboo spine” appearance. AS affects 0.5% of the global population. The creation of a rigid spinal column, with associated osteoporosis as the disease progresses, results in a brittle, rigid structure that is poorly suited to withstand stress. Thus, even minor trauma can result in a fracture [2–5].

Globally, the relative risk of traumatic vertebral fractures has been estimated to be three times higher in patients with AS than in the general population [4]. The cervical spine, especially the lower cervical spine and cervicothoracic junction, is the most common site of fractures in these patients. The diagnosis is difficult and thus may be delayed. The delay is partly attributed to the lack of pain associated with these fractures because (1) the patients are undergoing corticosteroid therapy for AS and (2) the preexisting spinal alterations. Plain radiography is inadequate for fully evaluating the shearing fractures due to osteoporosis and to the position of the shoulders [3, 6–9].

AS-related fractures are frequently more severe than cervical spinal fractures in an otherwise healthy population because of their specific features. For instance, AS fractures are highly unstable because of the anterior and the posterior elements that are involved. Moreover, the broken “bamboo spine,” resembling long-lever arms, is extremely unstable with a high risk of neurological deterioration. In addition, the kyphotic deformation does not provide the most appropriate sagittal balance for primary stability. Despite these considerations, these bones display a good propensity to fuse [8, 10].

Surgical treatment is controversial, with several reports having described an array of surgical techniques to treat these fractures and their resulting deformities [3, 5–8, 11]. The aim of this study was to show the surgical experience of treating lower cervical spine fractures in patients with AS using posterior pedicle screw fixation alone.

## Methods

### Patient population

The study is based on data from patients with AS and lower cervical spine fractures who underwent posterior cervical/thoracic pedicle screw fixation between 2006 and 2013. The study design is a retrospective chart review with radiological follow-up. The study group consisted of 35 patients (34 men, 1 woman; mean age 57 years, range 37 − 68 years). On average, AS had been diagnosed 21 years previously. The mechanism of the injury was of low energy in 25 cases, violent in 8 cases, and unknown in 2 cases (Table 1). On inspection, the patients had obvious preexisting thoracolumbar and cervicothoracic kyphosis, with markedly forward head positioning.

**Table 1:** Summary of patient’s demographic data

Variables	
Sex (F/M)	1/34
Age (mean, yrs)	57
Mechanism of injury	
Low-energy	25
High-energy	8
Unknown	2
Level of fracture	
C5–6	11
C6	5
C6–7	16
C7	3

All patients were evaluated with plain radiography, computed tomography (CT), and magnetic resonance imaging (MRI). CT scans were used to elucidate the bone detail of the fracture, deformity, and tissues surrounding the fixation site. MRI was used to differentiate the various causes of cord compression and to identify cord contusion. Two experienced spinal surgeons performed all of the radiological studies. The fracture levels were C5-6 in 11 patients, C6 in 5 patients, C6-7 in 16 patients, and C7 in 3 patients.

The neurological injury was evaluated based on the American Spinal Injury Association (ASIA) impairment scale. At admission, there was a high incidence of neurological deficit after the fracture. Among the 33 cases with neurological deficit, the deficit was reported as grade A in 5 cases, grade B in 4 cases, grade C in 12 cases, and grade D in 12 cases.

### Treatment and surgical techniques

Management was based on the presence or absence of spinal instability and neurological symptoms. In the presence of both instability and neurological deficit, surgery was performed to achieve stabilization and decompression. All 35 of our patients underwent posterior surgery alone. Posterior stabilization was achieved using pedicle screw fixation between C4 and T2.

Decompression of spinal stenosis may be performed during the same operative session. Patients with AS are at increased risk for posture deterioration and iatrogenic fractures of the spine during the surgical procedure, especially while under sedation/anesthesia. A possible detrimental consequence is worsening of the neurological impairment. To avoid further motion of the cervical spine in AS patients, an awake nasoendotracheal intubation should be performed blindly using a laryngeal mask to guide the tube.

Cervical pedicle screws are the most reliable—although the most challenging—type of fixation. A modified Abumi technique was used to place these screws. Using a lateral C-arm view and anatomical landmarks, a large hole was created in the lamina at the pedicle entrance, and the pedicle was gently probed before screw insertion. A definitive tapered rod, which tapered from 3.2 to 5.5 mm, was applied from the immobilized lower cervical vertebrae to T2. A pre-bending rod and distraction can be performed to increase the chin-to-chest distance gradually and restore the former lordosis. The rhythm and amount of correction must be performed under neurophysiological monitoring. Usually, postoperative immobilization is mandatory because of the specificities of those patients (i.e., residual kyphosis and sagittal imbalance, poor bone quality, highly unstable lesions). A custom-made cervicothoracic orthosis could be sufficient.

### Follow-up

Patients were monitored for neurological outcome, radiographic fusion, and complications. Complications were categorized as general (e.g., infection, dysphagia, death) or surgical (e.g., instrumentation failure) subtypes. Postoperative follow-up examinations were performed at intervals of 4 weeks, 3 months, 6 months, and annually thereafter, with radiographic evaluations including CT scans and sagittal reconstruction obtained immediately postoperatively and again at the 3-, 6-, and 12-month follow-up examinations. Fusion was considered complete when trabecular bone was visible and spanned the fracture site. Cervical deformation and correction of the pre-fracture kyphosis were evaluated by comparing the regional deformation immediately preoperatively, postoperatively, and/or at the last follow-up.

## Results

**Fig. 1:**
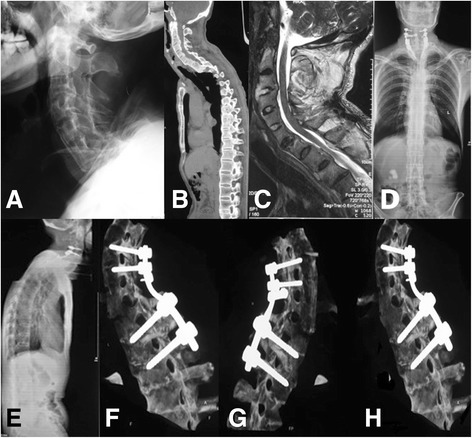
A 53-year-old man with ankylosing spondylitis and a complete fracture of C7 and partial dislocation. **a** Preoperative plain radiograph showing a displaced fracture together with an obvious gap in the anterior column at C7 because the rigid elevation of the shoulders prevents adequate imaging at the cervico-thoracic junction and so called bamboo spine resulting from ankylosing spondylitis; (**b**) and (**c**) CT with sagittal reconstruction and MR imaging showing an obvious fracture of C7. **d** and (**e**) Immediate antero-posterior and lateral radiographs showing posterior stabilization using pedicle screw fixation between C4 and T2. **f** and (**g**) Postoperative CT with sagittal reconstruction showing good screws placement in the cervical and thoracic pedicles, the cervicothoracic pre-existing kyphosis has been corrected. **h** Bone fusion has been confirmed by sagittal reconstruction CT after 6 months postoperatively

In our series, 32 (91.4%) of the 35 fractures were due to acute injuries, and 3 (8.6%) were chronic in nature. A single spinal segment was involved in each patient. The C6-7 level was the most frequently injured site (16 cases, 45.7%). A low-energy injury of the spine occurred in 25 cases and a high-energy injury in 8 cases. The mechanisms of the fractures were hyperextension in 19 patients, flexion in 12 patients, and compression in 2 patients. All 35 patients were treated successfully using posterior pedicle screw fixation (Fig. 1). Posterior decompression was performed in the patients with a neurological deficit.

The mean duration of the operation was 4.4 h (range 3.2 − 6.4 h). The average estimated blood loss was 740 ml (600 − 1200 ml). Instrumentation spanned four vertebrae: two cephalad and two caudal to the fracture. Altogether, 280 pedicle screws were placed: 32 in C4 pedicles, 70 in C5 pedicles, 38 in C6 pedicles, 70 in T1 pedicles, and 70 in T2 pedicles.

Two cervical pedicle screws were misplaced superiorly, and two were misplaced laterally in the pedicles. Three thoracic pedicle screws were misplaced slightly medially. Although all of the misplaced pedicle screws were evaluated using postoperative CT to determine their positions, none of the seven displaced screws induced any symptoms, and they did not require surgical extraction. In summary, 4 of the 140 cervical pedicle screws (2.9%) and 3 of the 140 thoracic pedicle screws (2.1%) were misplaced.

An average of 5.6 segments were instrumented in each case, with harvesting of iliac crest bone graft material in 10 patients (28.6%). All of the others received autogenous local bone graft obtained from the spinous processes and lamina.

Four patients died from unrelated causes during the postoperative follow-up period. Death was due to pneumonia in two of the patients, at 6 and 13 months, respectively. Intestinal obstruction occurred in one patient at 13 months postoperatively, and one patient suffered a heart attack at 4 months. The other 31 patients had a mean follow-up time of 26 months (range 11 − 48 months).

All patients achieved radiographic fusion, which was confirmed by CT. The average bone fusion time was 3.6 months (range 3 − 6 months). There was no hardware failure (e.g., broken screws, pullout, migration) during the follow-up period.

Before the operation, two (5.7%) patients were neurologically intact, and five (14.3%) patients had a complete cord injury. In all, 28 (80%) patients sustained an incomplete cord injury, with four (11.4%) patients at ASIA grade B, 12 (34.3%) at ASIA grade C, and 12 (34.3%) at ASIA grade D. Although the number of patients was limited, the patients’ neurological status, when influenced, had improved. Postoperatively, 15 (42.9%) patients had improved ASIA grades: two (5.7%) from grade B to grade C; eight (22.9%) from C to D; and five (14.3%) from D to E (Table 2).

**Table 2:** Patients’ neurological status before and after the operation according to American Spinal Injury Association (ASIA) impairment scale

Preoperatively		Neurological status	Postoperatively	
5	14.3%	ASIA A	5	14.3%
4	11.4%	ASIA B	2	5.7%
12	34.3%	ASIA C	6	17.1%
12	34.3%	ASIA D	15	42.9%
2	5.7%	ASIA E	7	20.0%

There were no intraoperative complications. One patient experienced a superficial wound infection, which was managed with daily wound changes and the use of local and systematic antibiotics. A cervical esophageal fistula occurred 3 days postoperatively in one patient. Reoperation was undertaken, and the fistula was repaired with a pedicled fascial patch. Follow-up included fasting and gastrointestinal nutrition. The patient recovered 2 weeks later.

Preexisting cervicothoracic kyphosis was corrected during the posterior fixation procedure. The average postoperative correction was 18° (range 12° − 32°). The correction did not result in neurological decompensation.

## Discussion

### Clinical characteristics of lower cervical spine fracture with AS

Patients with AS are at higher risk of vertebral fractures than the rest of the global population. A recent retrospective cohort study of more than 230,000 patients reported a 3.3 times greater risk of vertebral fractures in patients with AS than in healthy persons [4, 10]. This higher risk of fracture is related to: (1) the spinal rigidity in AS due to progressive bone bridges that ultimately incorporate posterior facet joints, ligaments, and intervertebral discs; (2) kyphotic deformation, leading to trauma during hyperflexion − hyperextension and reduction of the visual field; and (3) association of AS with osteoporosis (25% − 50% of patients with AS present with associated osteoporosis) [9, 12–15].

AS-related cervical spine fractures are frequently more severe, with specific features, than the corresponding fractures in the healthy population. First, osteoporosis and deformity lead to increased weakness of the spine. As a consequence, even minor trauma can cause fractures because of the spine’s poor elasticity. A recent review of the literature showed that 65% of 345 patients with a cervical spine fracture in a context of AS presented after minor trauma. In our series, minor trauma was responsible for the fracture in 25 patients. Two patients presented with a fracture unrelated to trauma.

AS-associated fractures are highly unstable because the anterior and posterior elements exhibit a transverse or short oblique pattern that does not follow the classic three-column criterion for stability, as is seen in normal spines. Moreover, the broken “bamboo spine” behaves somewhat like a long bone with a diaphyseal fracture. That is, the long-lever arms are extremely unstable, with an associated higher risk of neurological deterioration. In addition, the kyphotic deformation does not provide the most appropriate sagittal balance for primary stability, and the hemorrhagic trend produces a supplementary risk of neurological complications because of the increased possibility of a compressive epidural hematoma.

In our series, all fractures occurred in the lower cervical spine and were accompanied by dislocation [13, 16]. Overall, 26 patients had neurological signs at admission, and 7 patients presented with neurological deterioration prior to surgical treatment. In addition, there was a high incidence of respiratory complications. A previous study reported a mortality rate of 20% − 57%, which was twice as high as that for a cervical spine fracture in the general population [17]. One explanation may be that it is associated with a systemic disease, AS, which can result in pulmonary fibrosis and reduced lung capacity. Finally, the diagnosis is difficult and may be delayed. The delay could be partly attributed to (1) the lack of pain associated with these fractures as the patients are undergoing corticosteroid therapy, and (2) the preexisting spinal alterations. Standard radiography is inadequate for fully evaluating shearing fractures due to osteoporosis and the position of the shoulders. Some authors have advocated MRI for detecting traumatic lesions specifically at the cervicothoracic junction, which is poorly documented radiologically. At the very least, a CT scan is necessary to show the fracture line, which usually runs transversely or is shortly oblique and complete from the anterior to the posterior side, similar to the classic Chance fracture at the thoracolumbar spine [2, 10, 18].

### Treatment of the AS-associated lower cervical spine fracture

The definitive treatment for cervical spine fractures in AS patients remains controversial, with various treatment modalities and outcomes reported in the literature [4, 6, 12, 16, 19]. Conservative treatment has long been the gold standard. It typically involves bed rest, skull traction, and/or wearing a halo vest or a head − cervical chest cast. Bed rest, however, is not appropriate for these patients as they are susceptible to pulmonary and decubitus complications. Skull traction cannot satisfactorily reduce or stabilize the cervical spine because of its stiffness and the irreducible surrounding soft tissues. Neurological deterioration had been reported as a result of distraction at the fracture site while applying traction. Simple external fixation cannot eliminate movement between the fracture sites, leading to pseudoarthroses. Non-union, loss of reduction, and neurological deterioration have been reported after non-operative treatment, often leading to a second operation. According to recent publications and in our own experience, such treatment strategies are not to be recommended because of the associated long-term immobilization and to complications related to bed rest and skull traction.

**Fig. 2:**
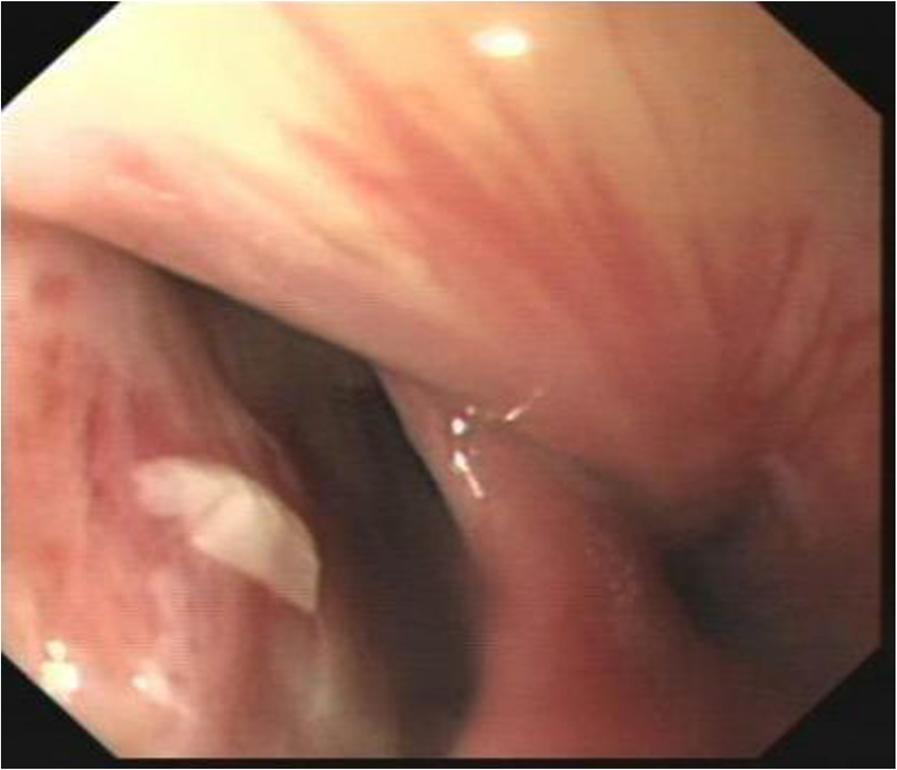
Gastroscopy performed three days postoperatively showing 1.5 cm × 1.5 cm circular fistula located at esophageal entrance posterior wall

The primary aim of surgical treatment is to maintain the fracture’s realignment with adequate stabilization measures until the bone has healed completely. Spinal stenosis may be decompressed during the same operative session. It may be achieved through a single anterior, single posterior, or combined one-time or staged posteroanterior or anteroposterior approach. The anterior approach has the benefit of short operative times, lower neurological deterioration due to positional changes, restoration of cervical alignment, and direct decompression. However, the poor results and high complication rate (cervical esophageal fistula, internal fixation failure) cannot be ignored. In AS patients, the esophageal mucosa is prone to injury due to ossification of the anterior longitudinal ligament and fracture displacement. Moreover, the anterior approach to the cervicothoracic junction is extremely difficult because of the great inclination and the kyphosis that exist in this region. The esophageal mucosa is prone to iatrogenic injury during the operation and to repeated friction by the anterior plate postoperatively. In our series, although not an anterior operation, a cervical esophageal fistula occurred within 3 days postoperatively in one case (Fig. 2). A possible cause was that the esophageal mucosa experienced gradual, repeated friction caused by progressive ossification of the anterior longitudinal ligament. After injury, the esophageal fistula was due to a shift of the sharp fracture end. The failure rate of anterior internal fixation is 50% because anterior implants are unstable and do not resist or absorb the tension from the posterior spinal column after three-column spinal injuries and because of the presence of severe osteoporosis. According to recent publications and in our own experience, anterior-only stabilization is rarely performed because of the aforementioned conditions.

Most authors have indicated that posterior fixation alone seems stable enough, provided that the number of fixations is sufficient (at least two above and two below the fracture). Cervical pedicle screws are reported to be the most reliable—but most challenging—type of fixation. The pull-out strength of various screw placement techniques in the cervical spine has been analyzed. Pedicle screws have been demonstrated to offer the best pull-out resistance of all available posterior fixation techniques, with an 88% increase in pull-out strength, compared with lateral mass screws [5, 13]. Given the anatomical bone distortion secondary to the underlying disease process, the typical landmarks are often obscured, making hardware placement a unique challenge in patients with AS. Detailed knowledge and familiarity with the lateral mass and pedicle anatomy are essential for extrapolation of limited recognizable landmarks during hardware placement and trajectory infiltration [18]. In our series, posterior pedicle screw fixation was completed successfully in all 35 patients, who had satisfactory clinical outcomes.

Because the cervical spine is kyphotic, the occurrence of the fracture could be an opportunity to increase lordosis at the site of the fracture. Most published series, however, have aimed only at restoring the former kyphosis [15, 17, 20]. In our experience, the pre-bending rod and distraction can be used to increase the chin-to-chest distance gradually and restore the former lordosis. This procedure did not increase the surgical duration. It is clear that the risk of translation and overdistraction is high during the lordosis maneuvers because of the instability. The anterior opening must be limited, specifically above C6, to avoid any additional cerebral risk of stretching the vertebral arteries. In our study, the correction did not result in neurological decompensation, and the average postoperative correction was 18°. All of the patients were satisfied with the results as their field of vision had improved.

There are several limitations in this study. First, the enrollment in this study was relatively small. As more cases are performed using this technique, however, its safety and efficiency can be more thoroughly evaluated. Second, we did not have controls to prove the outcomes. Prospective, controlled studies are currently being performed to meet this objective.

## Conclusions

Pedicle screw fixation and autologous bone graft through a single posterior approach for treating lower cervical spine fractures in AS patients can stabilize the spine, correct kyphosis, and relieve pressure. It is reasonable, therefore, to recommend this surgical strategy in patients with AS who have a fracture of the lower cervical spine.

## Abbreviations

3D-CT: Three-dimensional computed tomography
AS: Ankylosing Spondylitis
ASIA: American Spinal Injury Association
CT: Computed tomography
ICBG: Iliac Crest Bone Graft
MRI: Magnetic resonance imaging
